# Trends in Socioeconomic Inequalities in HIV Prevalence among Young People in Seven Countries in Eastern and Southern Africa

**DOI:** 10.1371/journal.pone.0121775

**Published:** 2015-03-20

**Authors:** James R. Hargreaves, Calum Davey, Elizabeth Fearon, Bernadette Hensen, Shari Krishnaratne

**Affiliations:** Department of Social and Environmental Health Research and Centre for Evaluation, London School of Hygiene and Tropical Medicine, London, United Kingdom; Karolinska Institute, SWEDEN

## Abstract

**Background:**

In Eastern and Southern Africa, HIV prevalence was highest among higher socioeconomic groups during the 1990s. It has been suggested that this is changing, with HIV prevalence falling among higher-educated groups while stable among lower-educated groups. A multi-country analysis has not been undertaken.

**Methods:**

We analysed data on socio-demographic factors and HIV infection from 14 nationally representative surveys of adults aged 15-24 (seven countries, two surveys each, 4-8 years apart). Sample sizes ranged from 2,408-12,082 (72,135 total). We used logistic regression to assess gender-stratified associations between highest educational level attended and HIV status in each survey, adjusting for age and urban/rural setting. We tested for interactions with urban/rural setting and age. Our primary hypothesis was that higher education became less of a risk factor for HIV over time. We tested for interaction between survey-year and the education-HIV association in each country and all countries pooled.

**Findings:**

In Ethiopia and Malawi, HIV prevalence was higher in more educated women in both surveys. In Lesotho, Kenya and Zimbabwe, HIV prevalence was lower in higher educated women in both surveys. In Ethiopia, HIV prevalence fell among no and secondary educated women only (interaction p<0·01). Only among young men in Tanzania there was some evidence that the association between education and HIV changed over time (p=0·07). Pooled analysis found little evidence for an interaction between survey year and the education-HIV association among men (p=0·60) or women (p=0·37).

**Interpretation:**

The pattern of prevalent HIV infection among young adults by level of education in different sub-Saharan African countries was heterogeneous. There was little statistical evidence that this pattern changed between 2003-5 and 2008-12. Explanations for the social epidemiology of HIV in Africa will need to account for time-trends and inter-country differences.

## Introduction

Socioeconomic inequalities in health in low and middle income countries remain poorly understood. HIV incidence remains unacceptably high throughout Eastern and Southern Africa [1]. Surveillance data to the mid-1990s showed that higher educational attainment and household wealth tended to be associated with higher HIV prevalence [2, 3]. This distribution is unusual compared with the social distribution of many health outcomes. However, it has previously been suggested that in the case of the emergence of a new infectious agent such as HIV it was perhaps to be expected that before 1996–2000, HIV would be more concentrated among those of higher socioeconomic position [4, 5]. HIV spread in sub-Saharan Africa through the mobility and size of sexual networks which are likely associated with greater wealth, and during the same period HIV prevention and treatment efforts were weak, awareness of HIV low and behaviour change largely absent [6].

This pattern may be changing over time [4, 7–9, 10–12]. For example, in Tanzania HIV prevalence fell faster among higher educated groups than among those with lower levels of education between 2003/4 and 2007/8 [6]. This may illustrate lower HIV incidence in higher educated groups between the two surveys, although cohort-effects, mortality and/or migration patterns might offer alternative explanations. In Tanzania, sexual behaviour patterns by educational attainment in 2003/4 and 2007/8 offered further support that HIV incidence patterns may have been a key driver of the HIV prevalence trends observed [13]. This pattern may be in line with “the inverse equity hypothesis” [14]. Developed originally in relation to child health, the hypothesis proposes that higher socioeconomic groups benefit first from the introduction of new public health interventions such as, in this case, the widespread delivery of HIV prevention interventions in sub-Saharan Africa, particularly since 2000 [14].

A multi-country analysis has not previously been undertaken. We analysed changes between 2003–2005 and 2008–2012 in HIV prevalence among differently educated groups across Eastern and Southern Africa. Our primary hypothesis was that higher education became less of a risk factor for HIV over time.

## Methods

We collated data on socio-demographic factors and HIV infection among 15–24 year olds from 14 nationally-representative population-based surveys from seven countries. The surveys were conducted in Ethiopia (2005; 2011), Kenya (2004; 2008), Lesotho (2004; 2009), Malawi (2004; 2010), Rwanda (2005; 2010), Tanzania (2003; 2011/12), and Zimbabwe (2005; 2010) [15–28]. We restricted our analysis to young people aged 15–24 years as patterns of HIV prevalence among this group should be closely related to patterns of HIV incidence. Countries were included if they were in eastern or southern Africa, and if individual level data on HIV infection, educational attainment, sex, age and urban/rural status were available from at least two publicly-available surveys. Educational attainment was measured in terms of highest level of education attended using three groups: none, primary and some secondary or more. All analyses were stratified by sex and accounted for the sampling strategy deployed in the surveys by using probability weighting.

Our analysis had five steps. First we described the populations of participants. Second, we assessed the association between education and prevalent HIV infection within each survey. We calculated HIV prevalence and associated 95% confidence intervals for the different educational groups, and present Wald p-values for the education parameter from logistic regression models controlling for age as a linear term and urban/rural setting, after we had judged that there was little evidence for statistical interaction between the association of interest and urban/rural status. Third, we pooled the data from the two surveys in each country and assessed whether the association between education and HIV changed over time in each country by fitting adjusted, country-specific logistic regression models for the association between education and HIV and fitting an interaction term between education and survey (first survey/second survey). We represent the changes in the association between education and HIV prevalence between the two surveys (on the x-axis) by plotting (on the y-axis) prevalence of HIV for the no education, primary education and secondary education groups for men and women. Fourth, to assess whether the association between education and HIV changed over time across the whole region, we pooled the data. We recoded the primary sampling units so that they were unique for each primary sampling unit in each country. We fit the same model as above, additionally controlling for country as a fixed-term, having first assessed the strength of evidence for a systematic difference between countries in the trend over time in the association between education and HIV by fitting a 3-way interaction term (education/survey/country) in the model. Fifth, we repeated this final analysis but including the full set of individuals aged 15–49 years and additionally fitting another three way interaction term (education/survey/age group) to assess whether the overall pattern we describe among 15–24 year olds was systematically different from that observed in 25–49 year olds.

## Results

A total of 72,135 individuals (range 2,408 to 12,082) were included in the analysis (Table 1). Within each country the two surveys were conducted four to eight years apart. All samples were predominantly rural (65.5%- 85.0%). HIV prevalence ranged from 1.2% (Ethiopia, 2011) to 17.1% (Lesotho, 2004). HIV prevalence fell in all countries over time. HIV prevalence was higher among young women than young men in all educational groups in all countries, with a small number of exceptions, including among young people with no education in Ethiopia (2011) (prevalence among males 0.8%, females 0.5%) and Tanzania (2012) (3.9% males, 2.8% females), those with primary education in Malawi (2010) (6.8% males, 5.5% females) and those with secondary education in Tanzania (2003) (10.6% males, 8.2% females). The proportion of young people with no education varied from <1% (Zimbabwe, both years) to 35.1% (Ethiopia, 2005), and fell over time in all countries. The proportion of young women with no education was greater than that for young men, except in Lesotho in both surveys and in Zimbabwe in the second survey. In the earlier surveys in Rwanda and Tanzania more than 75% of the population were classified in the middle “primary educated” grouping, with relatively small proportions with no or secondary education.

**Table 1:** Characteristics of participants aged 15–24 years in seven nationally-representative population-based surveys of HIV infection.

		**Ethiopia**	**Kenya**	**Lesotho**	**Malawi**	**Rwanda**	**Tanzania**	**Zimbabwe**	**Pooled**
		N (%)	N (%)	N (%)	N (%)	N (%)	N (%)	N (%)	N (%)
**Survey 1**	Year	2005	2004	2004	2004	2005	2003	2005	Survey1
**Individual survey response rate (all ages)**	Male	89%	86%	85%	86%	97%	91%	82%	
	Female	96%	94%	94%	96%	98%	96%	90%	
**Sex**	Male	2040 (444)	1239 (46.3)	1071 (44.3)	1031 (42.8)	2051 (44.9)	2054 (46.3)	3078 (45.5)	12533 (45.1)
	Female	2551 (55.6)	1436 (53.7)	1345 (55.7)	1377 (57.2)	2520 (55.1)	2379 (53.7)	3681 (54.5)	15252 (54.9)
**Age**	15–19	2559 (55.7)	1444 (53.9)	1387 (57.4)	1162 (48.3)	2461 (53.8)	2424 (54.7)	3753 (55.5)	15129 (54.5)
	20–24	2032 (44.2)	1234 (46.1)	1029 (42.6)	1246 (51.7)	2110 (46.2)	2009 (45.3)	3006 (44.5)	12656 (45.6)
**Education**	None	1612 (35.1)	188 (7.0)	125 (5.2)	177 (7.4)	503 (11.0)	673 (14.9)	31 (0.5)	3302 (11.9)
	Primary	1730 (37.7)	1670 (62.4)	1474 (61.1)	1652 (68.6)	3610 (79.0)	3390 (75.2)	1804 (26.7)	15252 (54.9)
	Secondary	1249 (27.2)	820 (30.6)	817 (33.8)	579 (24.0)	459 (10.0)	448 (9.9)	4924 (72.9)	9231 (33.2)
**Setting**	Urban	1346 (29.3)	768 (28.7)	501 (20.7)	378 (15.7)	1079 (23.6)	1167 (26.3)	2332 (34.5)	7531 (27.1)
	Rural	3245 (70.7)	1910 (71.3)	1915 (79.3)	2030 (84.3)	3492 (76.4)	3266 (73.7)	4427 (65.5)	20254 (72.9)
**HIV (%)**		72 (1.6)	140 (5.2)	413 (17.1)	224 (9.3)	105 (2.3)	297 (6.7)	841 (12.4)	2086 (7.5)
**Survey 2**	Year	2011	2008	2009	2010	2010	2012	2010	Survey 2
**Individual survey response rate (all ages)**	Males	89%	89%	95%	92%	99%	89%	86%	
	Female	95%	96%	98%	97%	99%	96%	93%	
**Sex**	Male	5185 (42.9)	1322 (42.8)	1449 (44.9)	2938 (47.5)	2638 (46.7)	3682 (45.1)	2702 (45.5)	19912 (45.0)
	Female	6897 (57.1)	1700 (56.3)	1782 (55.2)	3244 (52.5)	3016 (53.3)	4482 (54.9)	3237 (54.5)	24350 (55.)
**Age**	15–19	6727 (55.7)	1590 (52.6)	1808 (56.0)	3499 (56.6)	3047 (53.9)	4664 (57.1)	3261 (54.9)	24590 (55.6)
	20–24	5355 (44.3)	1432 (47.4)	1423 (44.0)	2683 (43.4)	2607 (46.1)	3500 (42.9)	2683 (45.1)	19672 (44.4)
**Education**	None	2687 (22.2)	191 (6.3)	90 (2.8)	243 (3.9)	271 (4.8)	733 (9.0)	33 (0.6)	4249 (9.6)
	Primary	6914 (57.2)	1727 (57.2)	1544 (47.8)	4266 (69.0)	3957 (70.0)	4446 (54.5)	1418 (23.9)	24254 (54.8)
	Secondary	2481 (20.5)	1104 (36.5)	1597 (49.4)	1673 (27.1)	1426 (25.2)	2985 (36.6)	4493 (75.6)	15759 (35.6)
**Setting**	Urban	3937 (32.6)	799 (26.4)	681 (21.1)	928 (15.0)	1027 (18.2)	1955 (24.0)	1882 (31.7)	11201 (25.3)
	Rural	8145 (67.4)	2223 (73.6)	2550 (78.9)	5254 (85.0)	4627 (81.8)	6209 (76.1)	4062 (68.3)	33061 (74.7)
**HIV (%)**	Primary	147 (1.2)	145 (4.8)	482 (14.9)	404 (6.5)	122 (2.2)	280 (3.4)	634 (10.7)	2214 (5.0)

Among young women there were statistically significant associations (p<0.05) between education and prevalent HIV infection in nine of the fourteen surveys, and borderline associations (0.05<p<0.1) in a further two surveys (Table 2). In Malawi and Ethiopia, HIV prevalence was higher in more educated groups in both surveys. In Lesotho, Kenya and Zimbabwe, HIV prevalence among young women was lower in the secondary educated groups than primary educated groups in both surveys, and the proportion of young people with no education was low. In Tanzania in 2003, HIV prevalence was higher among those with greater educational attainment, but this was not statistically significant. By 2011/12 HIV had fallen in all three education groups, and had fallen furthest among those with secondary education, but there remained little evidence of an association. In the earlier Rwanda survey, HIV was lowest among those with primary education and similar in the other two groups. HIV prevalence fell over time in all three groups, but least in the primary group so that by 2010 there was little evidence of any association between education and HIV prevalence. In only one country, Ethiopia, the country with the lowest HIV prevalence, was there strong evidence of interaction between survey year and the relationship between education and HIV prevalence among young women (p<0.01). The changes over time in Ethiopia were not as we predicted: HIV prevalence fell among both those with no education and secondary education, but not among the primary educated group.

**Table 2:** HIV prevalence among women across education groups.

	**Education (attended)**			
Country (Year)	**None**	**Primary**	**Secondary**	p-value*
	**HIV Prevalence**			
	**n/N (%, 95%CI)**	**n/N (%, 95%CI)**	**n/N (%, 95%CI)**	
Ethiopia (2005)	11/1121 (1.0)	15/864 (1.7)	28/566 (5.0)	0.00
OR (95% CI)	1.6 (0.6–4.8)	-	7.8 (3.0–20.5)	
Ethiopia (2011)	9/1915 (0.5)	64/3704 (1.7)	27/1278 (2.1)	0.03
OR (95% CI)	0.2 (0.1–0.3)	-	0.8 (0.6–1.8)	
Kenya (2004)	5/141 (3.6)	66/882 (7.5)	20/416 (4.8)	0.01
OR (95% CI)	0.4 (0.1–1.1)	-	0.6 (0.3–1.0)	
Kenya (2008)	8/164 (4.9)	64/958 (6.7)	21/578 (3.6)	0.07
OR (95% CI)	0.8 (0.3–1.8)	-	0.7 (0.3–1.3)	
Lesotho (2004)	4/17 (23.5)	167/816 (20.5)	94/512 (18.4)	0.00
OR (95% CI)	0.9 (0.2–3.4)	-	0.9 (0.6–1.2)	
Lesotho (2009)	2/9 (22.2)	141/781 (18.1)	162/992 (16.3)	0.00
OR (95% CI)	0.7 (0.1–4.0)	-	0.7 (0.6–1.0)	
Malawi (2004)	14/125 (11.2)	103/964 (10.7)	38/288 (13.2)	0.01
OR (95% CI)	1.0 (0.5–2.0)	-	1.2 (0.7–2.0)	
Malawi (2010)	18/176 (10.2)	125/2289 (5.5)	82/779 (10.5)	0.00
OR (95% CI)	1.8 (0.8–3.7)	-	2.1 (0.4–3.0)	
Rwanda (2005)	11/306 (3.5)	48/1963 (2.5)	11/251 (4.4)	0.03
OR (95% CI)	1.4 (0.7–2.7)	-	2.0 (1.0–4.1)	
Rwanda (2010)	4/173 (2.3)	44/2107 (2.1)	20/736 (2.7)	0.64
OR (95% CI)	1.5 (0.5–4.5)	-	1.5 (0.8–2.6)	
Tanzania (2003)	22/431 (5.1)	122/1681 (7.3)	22/267 (8.2)	0.30
OR (95% CI)	0.6 (0.3–1.2)	-	1.2 (0.6–2.3)	
Tanzania (2012)	15/529 (2.8)	98/2419 (4.1)	50/1534 (3.3)	0.44
OR (95% CI)	0.9 (0.5–1.8)	-	1.3 (0.8–2.2)	
Zimbabwe(2005)	3/22 (13.6)	140/956 (14.6)	365/2703 (13.5)	0.01
OR (95% CI)	0.9 (0.2–3.5)	-	1.1 (0.8–1.4)	
Zimbabwe (2012)	4/14 (28.6)	93/746 (12.5)	287/4277 (11.6)	0.08
OR (95% CI)	1.7 (0.5–5.5)	-	0.9 (0.7–1.2)	
Pooled early surveys	70/2163 (3.2)	661/8126 (8.1)	574/4963 (11.6)	< 0.00
	0.4 (0.3–0.6)	-	1.3 (1.1–1.6)	
Pooled later surveys	60/2980 (2.0)	629/13004 (4.8)	649/8374 (7.8)	< 0.00
	0.5 (0.3–0.8)	-	1.3 (1.1–1.8)	

Adjusted Odds Ratios and 95% confidence intervals for the association between HIV and education attendance using primary education as a baseline.

* p-value from Wald test of the overall association between education and prevalent HIV infection adjusted for age and urban/rural status; Tests for interactions between the relationship between HIV and education and urban/rural setting showed no significant results, except among women in Zimbabwe in the first survey year (p = 0.02). There was no evidence of an interaction between whether the surveys were early or late and the association between education and HIV in the pooled data (p = 0.37).

**Fig 1:**
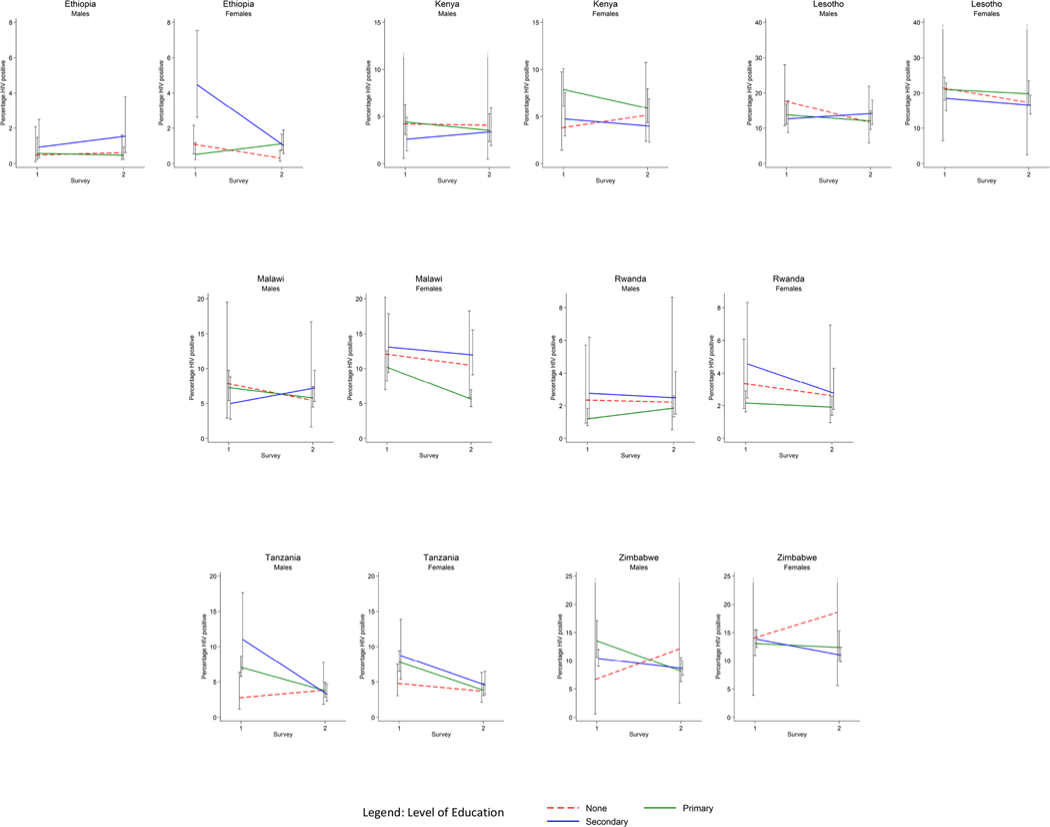
Changes in HIV Prevalence among men and women aged 15–24 years with different levels of educational attainment between earlier (2003–5) and later (2008–12) nationally-representative population-based surveys in seven sub-Saharan African countries.

Among young men there was little evidence of an association between education and HIV prevalence in either the first or second surveys, with two exceptions (Table 3). HIV prevalence was lowest in the primary educated group in Rwanda in 2010, where this group comprised 70% of the population. Among young men in Tanzania in 2003 there was some evidence of an upward gradient in HIV infection by educational status. This gradient was not present in 2011, and there was moderately strong evidence for an interaction between survey year and the relationship between education and HIV prevalence (Fig. 1). In line with our a priori hypothesis, HIV prevalence increased among Tanzanian young men with no education, was stable among those with primary education and fell among those with secondary education.

**Table 3:** HIV prevalence among men across education groups.

	**Education (attended)**			
**Country (Year)**	**None**	**Primary**	**Secondary**	**p-value** *
	**HIV Prevalence**			
	**n/N (%)**	**n/N (%)**	**n/N (%)**	
Ethiopia (2005)	2/491 (0.4)	7/866 (0.8)	9/683 (1.3)	0.31
OR (95% CI)	0.8 (0.2–4.4)	-	1.7 (0.4–8.1)	
Ethiopia (2011)	6/772 (0.8)	20/3210 (0.6)	21/1203 (1.8)	0.20
OR (95% CI)	1.6 (0.5–5.3)	-	5.3 (1.2–24.1)	
Kenya (2004)	1/48 (2.1)	37/788 (4.7)	11/403 (2.7)	0.76
OR (95% CI)	0.9 (0.1–7.3)	-	0.7 (0.3–1.4)	
Kenya(2008)	1/28 (3.6)	33/768 (4.3)	18/526 (3.4)	0.69
OR (95% CI)	1.5 (0.2–12.2)	-	1.1 (0.6- 2.1)	
Lesotho (2004)	16/108 (14.8)	98/658 (14.9)	34/305 (11.2)	0.65
OR (95% CI)	1.4 (0.8–2.5)	-	1.0(0.6–1.5)	
Lesotho (2009)	9/81 (11.1)	92/763 (12.1)	76/605 (12.6)	0.23
OR (95% CI)	0.9 (0.4–2.0)	-	1.2 (0.8–1.7)	
Malawi (2004)	5/52 (9.6)	51/688 (7.4)	13/291 (4.5)	0.65
OR (95% CI)	1.0 (0.3–3.2)	-	0.8 (0.3–1.7)	
Malawi (2010)	3/67 (4.5)	114/1977 (5.8)	62/894 (6.9)	0.26
OR (95% CI)	1.1 (0.3–3.6)	-	1.4 (0.9–2.2)	
Rwanda (2005)	5/196 (2.6)	24/1647 (1.5)	6/208 (2.9)	0.17
OR (95% CI)	1.9 (0.7–5.3)	-	2.3 (0.8–6.1)	
Rwanda(2010)	2/98 (2.0)	35/1850 (1.9)	17/690 (2.5)	0.00
OR (95% CI)	1.5 (0.3–6.3)	-	1.5 (0.8–2.7)	
Tanzania (2003)	6/234 (2.6)	105/1632 (6.4)	20/188 (10.6)	0.09
OR (95% CI)	0.4 (0.2–1.2)	-	1.7 (0.9–3.2)	
Tanzania (2012)	8/204 (3.9)	67/2027 (3.3)	42/1451 (2.9)	0.65
OR (95% CI)	1.0 (0.4–2.6)	-	0.8 (0.5–1.3)	
Zimbabwe (2005)	1/10 (10.0)	104/847 (12.3)	228/2221 (10.3)	0.13
OR (95% CI)	0.5 (0.1–3.9)	-	1.6 (0.3–8.2)	
Zimbabwe (2010)	2/19 (10.5)	63/672 (9.4)	185/2016 (9.2)	0.92
OR (95% CI)	0.7 (0.5–1.0)	-	1.0(0.7–1.4)	
Pooled	36/1139 (3.2)	426/7126 (6.0)	319/4268 (7.5)	0.26
	0.8 (0.5–1.2)	-	1.1 (0.9–1.3)	
Pooled	31/1269 (2.4)	424/11267 (3.8)	421/7385 (5.7)	0.12
	0.8 (0.5–1.3)	-	1.2 (1.0–1.6)	

Adjusted Odds Ratios and 95% confidence intervals for the association between HIV and education attendance using primary education as a baseline.

* p-value from Wald test of the overall association between education and prevalent HIV infection adjusted for age and urban/rural status; Tests for interactions between the relationship between HIV and education and urban/rural setting showed no significant results, except among women in Zimbabwe in the first survey year (p = 0.02). There was no evidence of an interaction between whether the surveys were early or late and the association between education and HIV in the pooled data (p = 0.6).

Fig. 1 shows changes in HIV Prevalence among men and women aged 15–24 years with different levels of educational attainment between earlier (2003–5) and later (2008–12) nationally-representative population-based surveys in seven sub-Saharan African countries. ‘No education’ is shown with a dotted red line, ‘Some primary education’ is shown with a solid green line, and ‘Some secondary education’ is shown with a solid blue line. Prevalence is % of the population who are living with HIV. Two different time points, one for survey 1 and one for survey 2, are shown on the X-axis. 95% confidence intervals are demonstrated for each prevalence.

When data from all the surveys was pooled, contrary to our hypothesis there was little evidence of an interaction by survey year in the association between education and HIV prevalence among either the men (p = 0.16) or women (p = 0.55). Further there was relatively weak evidence that this interaction term differed systematically between countries (p = 0.48 for men; p = 0.10 for women). We repeated this final analysis including men and women aged 25–49 years (n = 133, 247) and found little evidence that the trends by education observed in young men and women differed from that seen older men and women (p = 0.30 for men; p = 0.68 for women).

## Discussion

We analysed the association between education and HIV prevalence and whether this changed over time between 2003–5 and 2008–12 among young people aged 15–24 years in population-based, nationally-representative surveys in seven east and southern African countries.

**Fig 2:**
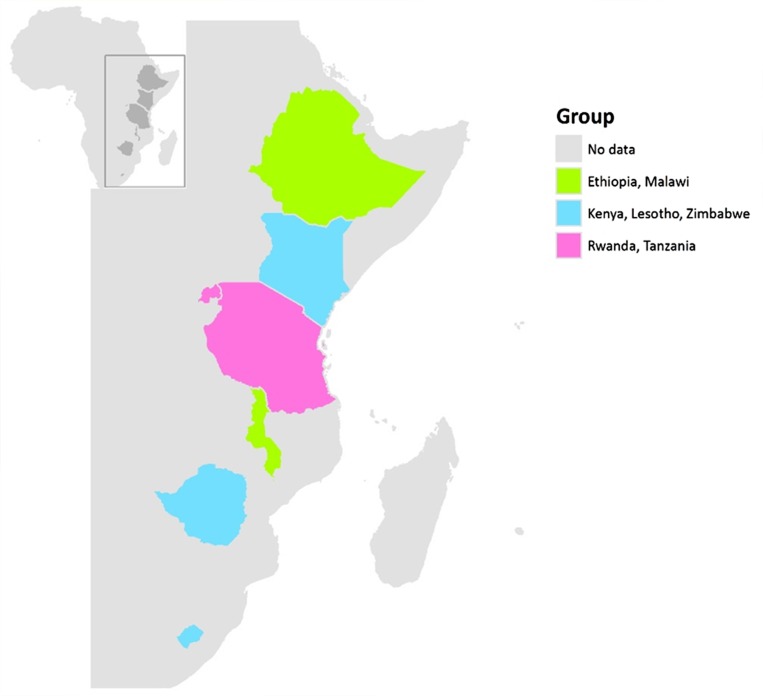
Geographic variation in the association between educational attainment and HIV prevalence among young women and how this changed between 2003–5 and 2008–12 in seven countries.

We identified three typologies in the association between education and prevalent HIV infection among young women (Fig. 2). Malawi was the most rural of the countries studied, while Ethiopia had the highest proportion of individuals with no education. In both these settings HIV prevalence was highest among young women with greater levels of education in both surveys. In Zimbabwe, Lesotho and Kenya, countries with relatively well-developed educational systems HIV prevalence was lower among the most educated young women in both surveys. In Rwanda and Tanzania the pattern was mixed. Among young men, HIV prevalence was generally lower than among women and there was little evidence of association with education. There were some small increases in HIV prevalence among males with secondary education in some countries. This differs from the consistent downward trend in HIV prevalence among women with more education across all countries. While these increases among men were very small, there may have been confounding factors that we did not capture within our analyses which may have influenced the findings. A limitation of our present analysis is a lack of data on potential confounders.

Fig. 2 demonstrates the geographic variation in the association between educational attainment and HIV prevalence among young women and how this changed between 2003–5 and 2008–12 in seven countries. Countries in green showed a higher HIV prevalence among more educated young women in both earlier and later surveys (Ethiopia, Malawi). Countries in blue showed a lower HIV prevalence among more educated young women in both earlier and later surveys, in countries with a low prevalence of no education (Lesotho, Kenya, Zimbabwe). Countries in pink showed no association between education and HIV in at least one of the surveys (Rwanda, Tanzania).

While we expected the distribution of prevalent infections in the earlier surveys to be heterogeneous, we hypothesized that a common pattern, predicted by existing theories of heath inequality, may be seen in changes over time. We suggested the association between HIV prevalence and having no education relative to the primary educated group would either increase towards the null or become a stronger risk factor between the first and second surveys, and that the equivalent association for higher educated groups relative to the primary educated group would move closer to the null or become more protective. Taken together the data were not supportive of this hypothesis. The patterns of change over time were not as we predicted, except among young men in Tanzania between 2003/4 and 2011/12. Among young Ethiopian women HIV prevalence fell among both those with no education and secondary education, but not among the primary educated group. Among the Tanzanian women, Ethiopian men and individuals of both sexes from Kenya, Lesotho, Malawi, Rwanda, and Zimbabwe there was little evidence that prevalence changes over time differed by educational attainment. The hypothesis that HIV patterns are changing over time universally across sub-Saharan Africa is not supported by this analysis and we feel that this is an important finding. It is unlikely that looking at absolute differences in HIV prevalence would have provided any more support for the inverse equity hypothesis.

We pooled data from over 70,000 young people who participated in high-quality, population-based, nationally-representative surveys making this the largest and most broadly representative investigation of this subject to date. However, limitations at several levels must be considered in interpreting our findings.

We analysed data from large, well conducted surveys undertaken with highly overlapping, though locally adapted, methodology. The demographic and health survey data are standardized and pre-tested to allow for comparability across populations and over time [29]. Household and individual survey response rates are high [30, 31]. HIV-testing response rates are generally lower than survey response rates and differ by urban/rural status, sex, education and socioeconomic group, with HIV-testing non-response generally higher among individuals of higher education and wealth [30]. Response rates were generally higher among women than men. The highest response rate was among both men and women in Rwanda. The overall response rate ranged from a low of 59.8% among men aged 15–19 years in Malawi in 2004 to 98.9% among men and women aged 15–19 years in Rwanda in 2010. HIV-testing non-response was, however, also high among some groups with no education. If HIV testing non-response is associated with HIV status then prevalence estimates may be biased [30]. Studies have shown that survey and HIV-testing non-responders often have higher predicted prevalence than responders; yet adjusting for non-response generally has non-significant effects on HIV prevalence estimates [30–32]. These analyses are subject to limitations as predicted prevalence is estimated based on imputation using the observed characteristics of individuals interviewed but refusing HIV-testing and using limited household questionnaire data for individuals refusing both the survey and HIV-testing [30, 32–34]. Although HIV prevalence among groups with no or secondary education may be underestimated in the surveys and need to be interpreted with caution, we suggest any bias is unlikely to alter our conclusions.

We interpreted HIV prevalence changes over time as a proxy for HIV incidence patterns during the period between the surveys. Nationally representative data on HIV incidence are not available for any of the countries included in this analysis. However, HIV prevalence changes over time are also influenced by patterns of mortality and migration. HIV mortality is strongly affected by time since infection and access to antiretroviral treatment. In many countries, available evidence suggests those of higher education were at greater risk earlier in the epidemic. Consequently, by the later surveys HIV-infected individuals from higher educational groups would on average have been infected for longer and may have been more likely to die, thereby contributing to a lower prevalence in these groups than would have otherwise been the case. Conversely, higher education individuals may have been more likely to access treatment and thus have had lower mortality levels than less educated individuals. The balance of these dynamics is not well understood. Better data on these phenomena and the better integration of socioeconomic stratification within mathematical models of HIV epidemiology could usefully contribute to understanding these relationships. A strength of our investigation was that we focused on young people, among whom mortality levels should be low and infections acquired recently and among who prevalence trends should therefore be more representative of incidence patterns. However, we also explored trends by education in individuals aged ≥25 years, and found little evidence that trends observed among younger women and men differed from those in older age groups.

A further weakness of our analysis is that we were not able to include data on access to HIV prevention interventions, or on determinants of HIV infection such as sexual behaviour. Our previous analysis of data from Tanzania suggested that riskier sexual behaviour was more commonly reported by those of lower education in both 2003/4 and 2007/8 [13]. Differences in access to and uptake of HIV prevention services across countries may have contributed to differences in HIV prevalence in across the different countries. In Tanzania, uptake of HIV testing also appears to have risen from low levels in all groups to more common access among those of higher socioeconomic position [20, 35].

Our investigation was motivated by considering whether empirical trends could be understood in terms of existing theories of health inequality [14]. We hypothesised that patterns of change over time observed in Tanzania, South Africa and in smaller studies in several other countries may be in line with the “inverse equity hypothesis” and may be widely seen [6, 36–39]. Socioeconomic differences in accessing effective health interventions are key drivers of health inequalities [40]. After 2000, HIV prevention interventions (principally behaviour change efforts, STI control and attempts to increase access to condoms) expanded rapidly across sub-Saharan Africa [41]. We hypothesised these may have been accessed more effectively by more educated, higher socioeconomic groups, and that consequently a consistent pattern would be that HIV prevalence would be expected to decline faster than among less educated groups. In line with previous reports, we identified different patterns in the social epidemiology of HIV infection across countries in the region [42, 43]. However, outside of the young Tanzanian men we found little evidence of the dynamic trends we had predicted.

Despite the size of our analysis, the data shown here only provide a window on the epidemic from 2003–5 to 2008–12. This relatively short period coincides with population—level HIV prevalence and incidence decline in all the countries studied [41]. Perhaps the period under study was too short to identify the trends we sought to investigate, or perhaps the critical window for changes in HIV prevalence associated with education happened prior to 2003. Alternatively, perhaps during the period examined in these data, lower socioeconomic groups were benefitting as much as higher socioeconomic groups from HIV prevention efforts. The inverse equity hypothesis suggests that with time lower socioeconomic groups begin to benefit from interventions, and that a threshold for benefit to the higher socioeconomic groups, and ultimately the whole population, is reached [14]. This will require further study in order to appropriately plan HIV prevention efforts going forward.

It is also possible that the particular history of HIV/AIDS as a public health problem in sub-Saharan Africa means that the inverse equity hypothesis does not hold for HIV prevention. The HIV epidemic has been unusual in the extent to which discourse about its epidemiology has been linked to poverty and inequalities. Furthermore, the unprecedented political and financial support to the HIV response relative to other public health concerns also makes it unusual [44],[45]. Inter-country differences might be due to differences in wealth and access to health care services as well as with the history, culture, religion and social norms. Given this, we appreciate that the insight gained from conducting a multi-country analysis such as this is that trends in HIV progression must be interpreted in light of such heterogeneity. Continued monitoring of the changing social epidemiology of HIV in Africa remains critically important to build and test theory and help guide ongoing efforts to accelerate equitable HIV incidence decline.
